# Dissolution of Platinum Single Crystals in Acidic Medium

**DOI:** 10.1002/cphc.201900866

**Published:** 2019-11-08

**Authors:** Daniel J. S. Sandbeck, Olaf Brummel, Karl J. J. Mayrhofer, Jörg Libuda, Ioannis Katsounaros, Serhiy Cherevko

**Affiliations:** ^1^ Helmholtz-Institute Erlangen-Nürnberg for Renewable Energy (IEK-11) Forschungszentrum Jülich GmbH Egerlandstr. 3 91058 Erlangen Germany; ^2^ Department of Chemical and Biological Engineering Friedrich-Alexander-Universität Erlangen-Nürnberg Egerlandstr. 3 91058 Erlangen Germany; ^3^ Interface Research and Catalysis, Erlangen Catalysis Resource Center Friedrich-Alexander-Universität Erlangen-Nürnberg Egerlandstr. 3 91058 Erlangen Germany

**Keywords:** fuel cells, platinum, platinum dissolution, platinum single crystals, stability.

## Abstract

Platinum single crystal basal planes consisting of Pt(111), Pt(100), Pt(110) and reference polycrystalline platinum Pt(poly) were subjected to various potentiodynamic and potentiostatic electrochemical treatments in 0.1 M HClO_4_. Using the scanning flow cell coupled to an inductively coupled plasma mass spectrometer (SFC‐ICP‐MS) the transient dissolution was detected on‐line. Clear trends in dissolution onset potentials and quantities emerged which can be related to the differences in the crystal plane surface structure energies and coordination. Pt(111) is observed to have a higher dissolution onset potential while the generalized trend in dissolution rates and quantities was found to be Pt(110)>P(100)≈Pt(poly)>Pt(111).

## Graphical abstract

**The electrochemical dissolution** of Pt is investigated in situ for well‐defined, low‐index single‐crystal facets, revealing transient trends.

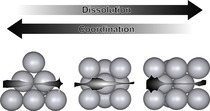

## 1 Introduction

In recent years, polymer electrolyte membrane fuel cell (PEMFC) performance has improved remarkably.^1^, ^2^, ^3^ With these improvements, PEMFCs now have the potential to obtain a large share of a growing electromobility market. However, cost and lifetime of PEMFC stacks still remain large challenges to be overcome.^4^ The majority of cost comes from the use of the precious metal platinum as a catalyst, which is dispersed as nanoparticles on a carbon support. Major degradation of the PEMFC stack can be attributed to PEM thinning, corrosion of the catalyst layer (CL) carbon support, and Pt dissolution. A PEMFC is expected to withstand hundreds of thousands of load cycles and tens of thousands of start‐up/shut‐down cycles during its lifetime, resulting in an enormous number of platinum oxidation/reduction cycles leading to extensive degradation.^3^ Degradation of CL can be further classified into several, sometimes interrelated mechanisms, namely agglomeration, reshaping, particle detachment, Ostwald ripening and dissolution.^5^, ^6^

Recent research on potential dependent transient dissolution of polycrystalline and carbon supported platinum has shed light on the nature of this degradation mechanism.^6^, ^7^, ^8^, ^9^, ^10^, ^11^, ^12^, ^13^, ^14^, ^15^, ^16^, ^17^, ^18^ The major results have been the elucidation of anodic and cathodic dissolution triggered by surface oxide formation and reduction, respectively. Additionally, the effects of temperature, reactive atmosphere, electrolyte composition, pH and Pt loading were further investigated. In the quest to improve electrocatalyst activity towards the oxygen reduction reaction (ORR) and reduce the required quantities of platinum in the CL, one promising strategy is the use of platinum and platinum alloy nanoparticles with preferential shapes exposing the low index face‐centered cubic crystal facets.^19^, ^20^, ^21^, ^22^, ^23^, ^24^, ^25^, ^26^, ^27^ Although activity trends have been identified among the varied shapes and exposed facets, reaching firm conclusions has required the development of techniques to produced very clean surfaces. These difficulties highlight the need for well‐defined systems in a bottom up approach to understanding electrocatalysis. Therefore, the dissolution behaviour of platinum basal surfaces remains to complete the fundamental picture necessary to unravel the intricacies of platinum nanoparticle stability.

Since the pioneering work of Clavilier, Conway and Jerkiewicz (among others),^28^, ^29^, ^30^ platinum single crystal electrochemistry has remained a prominent topic among researchers leading to many advancements in the last several decades, although often ripe with controversy.^31^ However, platinum single crystal electrochemistry remains a highly active field of research, including electrocatalytic activity studies,^32^, ^33^, ^34^, ^35^ investigations on improved preparation methods^36^, ^37^, ^38^, ^39^ and the use of advanced in situ characterization techniques to understand surface oxide formation and reduction.^40^, ^41^, ^42^ Despite this recent interest, very little literature is currently available on platinum single crystal transient dissolution.^43^, ^44^, ^45^

Most notable in Pt single crystal transient dissolution has been the recent works of Lopes et al.^44^, ^45^ Using the technique referred to as a stationary probe rotating disk electrode coupled to an inductively coupled plasma mass spectrometer (SPRDE‐ICP‐MS), key aspects of dissolution on the platinum single crystal basal planes were observed. The effect of reaction conditions was explored for the surfaces of Pt(111), Pt(100) and Pt(110) in 0.1 M HClO_4_ electrolyte.^44^ During CO stripping (oxidation) the trend in activity was found to be Pt(100)>Pt(110)>Pt(111), and dissolution was in the order of Pt(110)≫Pt(100)≫Pt(111). The activity trend for the ORR was found to be Pt(110)≈Pt(111)≫Pt(100) when cycling to 1.0 V_RHE_, while only a small amount of dissolution for Pt(110) was detected, as to be expected based on the limited surface oxidation at this potential. For the oxygen evolution reaction (OER) a clear activity‐stability relationship was observed, in which the activity trend was Pt(100)>Pt(110)≈Pt(111) while dissolution followed Pt(100)>Pt(110)>Pt(111). In an additional work focused on Pt(111), it was shown that anodic dissolution is independent of sweep rate, while cathodic is dependent.^45^ It was then suggested that anodic dissolution is solely an electrochemical process, while cathodic dissolution contains contributions from a chemical dissolution process.

The study presented here focuses on the transient dissolution of these surfaces under varying potentiostatic and potentiodynamic conditions using the in situ scanning flow cell coupled to an inductively coupled plasma mass spectrometer (SFC‐ICP‐MS),^9^, ^46^ which allows for the downstream detection of dissolution products. In contrast to the earlier work, which investigated Pt dissolution of the crystals during commonly studied electrochemical reactions (i. e. presence of various reactive gasses),^44^, ^45^ here the electrochemical conditions were systematically varied in inert Ar saturated 0.1 M HClO_4_ electrolyte. The HClO_4_ electrolyte was chosen for comparison to the earlier works on single crystal dissolution^44^, ^45^ and oxidation,^41^, ^42^ as well as many activity studies due to its non‐complexing nature.^19^, ^21^, ^23^, ^24^, ^25^, ^26^ The Pt basal planes, namely Pt(111), Pt(100), Pt(110) and also polycrystalline Pt(poly) were subjected to cyclic voltammetry (CV) with various upper potential limits (UPLs) and potentiostatic holds of varied potentials and time lengths. Clear surface dependent trends emerge in terms of total dissolved quantities and dissolution onset potentials which correlate to the intrinsically different atomic environments.

## 2 Results

### 2.1 Electrochemical Profiles of Platinum Single Crystals

**Figure 1:**
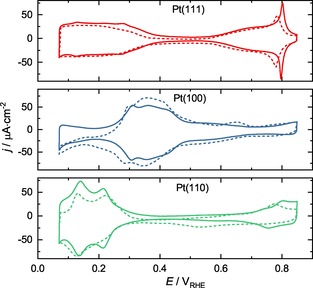
The characteristic CVs of the crystals taken in a bulk glass cell (solid) and SFC‐ICP‐MS (dashed) at 50 mV⋅s^‐1^ in 0.1 M HClO_4_

The quality of single crystals surfaces prepared by annealing and dipping in water was first confirmed by performing electrochemical experiments in a glass bulk cell. The characteristic features of the single crystal surfaces are clearly visible (solid lines in Figure 1), confirming a clean electrolyte solution and that the preparation procedure results in highly‐ordered surface structures. Next, the experiments were repeated in the scanning flow cell (SFC) setup. Comparing to the CVs taken in the bulk glass cell, the current densities are slightly diminished and some changes in the CV profiles are identified, which is likely due to small amounts of contaminations in this setup with increased complexity. However, we believe this to have a minimal impact on irreversible oxide formation and reduction and the subsequent dissolution, as has been previously suggested,^43^ while contaminations≥300 ppm typically cause severe changes in the CVs.^44^, ^47^, ^48^, ^49^, ^50^ Furthermore, the high reproducibility of the dissolution experiments suggests relatively clean surfaces.

### 2.2 Dissolution: Cyclic Voltammetry

**Figure 2:**
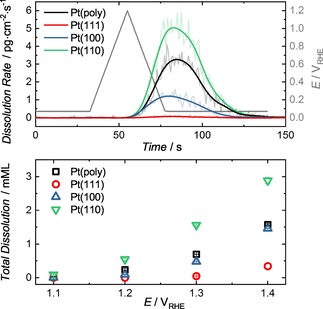
Example of transient dissolution profile of the studied working electrodes during a single cyclic voltammogram from 0.07–1.2 V_RHE_ at 50 mV ⋅ s^−1^ (top). Total quantity of Pt dissolved during a single CV to varying UPLs (bottom).

Dissolution of the crystals was measured during single CV scans to varying upper potential limits (UPLs) always with a freshly annealed, well‐ordered electrode. An example of the observed transient dissolution measured via the SFC‐ICP‐MS is illustrated in Figure 2 with the resulting total quantity dissolved calculated via integration of the dissolution peaks. Dissolved quantities are expressed in mMLs to account for varying surface atomic densities among the basal planes and Pt(poly), and can thus be considered an optimal metric of normalization. For reference, an analogous plot of dissolved quantities in mass per area, i. e. ng ⋅ cm^−2^, is shown in Figure S1.

It can be seen that dissolution increases with increasing UPL, and that the trend between the different crystals correlates to the differences in surface energy^51^ and surface atom coordination.^44^ Pt(111) is the most stable towards dissolution, which can be expected based on the lower surface energy relative to Pt(100), Pt(110) and Pt(poly). No significant dissolution of Pt(111) was detected during CVs with UPL≤1.2 V_RHE_, which can be expected based on previously reported stability during potential sweeps to these potentials.^45^, ^52^ In general the trend in total quantity dissolved within a single cycle is Pt(110)≫Pt(poly)≈Pt(100)≫Pt(111).

### 2.3 Anodic Dissolution: Onset Potential

**Figure 3:**
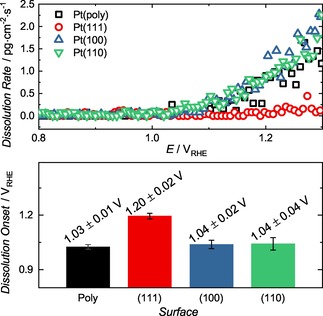
Dissolution of the studied working electrodes during an anodic sweep at 10 mV ⋅ s^−1^ (top) and estimated onset potentials of anodic dissolution (bottom).

The onset potential of anodic dissolution was estimated via linear potential sweeps at 10 mV ⋅ s^−1^. Figure 3 demonstrates the detected onsets of dissolution and the average values obtained. Examples of the data treatment for onset estimation are shown in Figure S2. Pt(111) has the largest onset potential of 1.20±0.02 V_RHE_ while Pt(100), Pt(poly) and Pt(110) behave similarly and are dissolved at approximately 150–200 mV more negative potentials. The onset of cathodic dissolution is difficult to estimate during such experiments, given the resolution of the dissolution rate and overlap of the anodic and cathodic peaks (Figure S2). Considering cathodic dissolution depends on oxide formation, electrochemical protocols should also be chosen carefully to elucidate this phenomenon. Future investigations will require further system development and dedicated experimentation to further probe this aspect of low index facet dissolution.

### 2.4 Dissolution: Potentiostatic Holds

**Figure 4:**
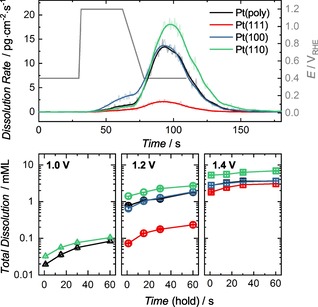
Example of transient dissolution profile of the studied working electrodes during potential hold experiments (top). Total quantity of Pt dissolved during potential hold experiments to varying UPLs (identified on the graphs) and time lengths (bottom).

In order to resolve the separate processes of anodic and cathodic dissolution, potential hold experiments were conducted for varying lengths of time and UPLs. Figure 4 shows an example of the measured transient dissolution, for an experiment with a hold time of 30 s, and the total quantities dissolved for all experiments. Dissolution rates and dissolved quantities increase with increasing UPL and with increasing time. The trend in total quantity dissolved between the different surfaces is Pt(110)>Pt(poly)≈Pt(100)>Pt(111) for UPLs 1.4 V and 1.2 V, while for UPL 1.0 V the trend is Pt(110)>Pt(poly)>Pt(100)>Pt (111). No dissolution of Pt(111) was detected for experiments of UPL 1.0 V and only 5 μML was detected for Pt(100) during 60 s at this potential. Dedicated plots for each potential are shown in Figure S3 and Figure S4 in units of dissolved mMLs and also ng ⋅ cm^−2^. Trends in mass per area deviate slightly in some experiments, due to the differences in atomic surface density.

**Figure 5:**
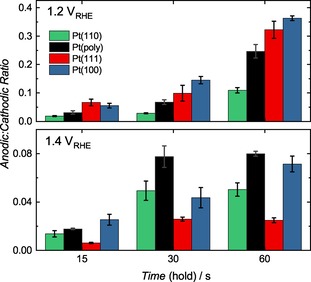
The ratio of Pt dissolved anodically and cathodically during potential hold experiments at 1.2 V_RHE_ and 1.4 V_RHE_ for 15, 30 and 60 s.

The difference between anodic and cathodic dissolution is represented in Figure 5 as the ratio of the total quantity dissolved anodically during the potential hold, and total quantity dissolved during the subsequent cathodic sweep (total dissolved quantities for the separate anodic and cathodic processes are shown in Figure S5). The 1 s hold experiments are omitted as such short time scales at anodic potentials render such a treatment impossible. Clearly, large differences exist between the different surfaces, which should be expected for inherently different oxidation, reduction and subsequent dissolution mechanisms, with a trend of Pt(100)>Pt(111)>Pt(poly)>Pt(110) for holds at 1.2 V_RHE_. At 1.2 V_RHE_ anodic dissolution is relatively most significant for Pt(111) and Pt(100), while cathodic dissolution for Pt(110) is clearly more severe. Pt(poly) has an intermediate ratio, which could be expected for a surface composed of a mixture of different crystallographic planes. At 1.4 V_RHE_ the trend does not persist, where here Pt(poly)>Pt(100)>Pt(110)>Pt(111).

For all surfaces, the anodic/cathodic ratio increases with increasing hold time, due to the more drastic increase in anodic dissolution (Figure S5). Anodic dissolution slightly decreases at the higher UPL of 1.4 V_RHE_, likely due to accelerated formation of passivating oxide films, while cathodic dissolution increases due to this increased oxide formation (and subsequent reduction).

## 3 Discussion

It has been shown that the trend in dissolution during CVs follows Pt(110)≫Pt(poly)≈Pt(100)≫Pt(111), and for potential holds at UPL≥1.2 V Pt(110)>Pt(poly)≈Pt(100)>Pt(111) and for potential holds at UPL 1.0 V_RHE_ Pt(110)>Pt(poly)>Pt(100)>Pt(111). In general, these trends among the single crystal basal planes could be rationalized by reported surface energies, where DFT calculated values typically follow Pt(110)>Pt(100)>Pt(111).^51^, ^53^ This reflects the fact that Pt(111) is a much easier surface to work with than Pt(110) and Pt(100), which readily undergo reconstruction.^36^ However, this correlation should be taken with a high degree of caution, considering large discrepancies in surface energies exist between theoretical calculations, while experimental values are unavailable. Furthermore, calculated bare (in vacuum) surface energies do not consider the impact of adsorbed species, water, electrolyte ions and surface polarization.

Surface energy is a thermodynamic quantity and also may not necessarily reflect transient, dynamic processes. The platinum basal planes inherently become more “open” when going from Pt(111), Pt(100) to Pt(110). Specifically, the topmost layer of atoms have different coordination numbers between these facets, in the order of 7, 8 and 9 for Pt(110), Pt(100) and Pt(111), respectively. Similar defect sites exposing undercoordinated Pt atoms are known to drastically alter adsorption properties and electrocatalytic activity. Not only should this also effect the thermodynamics of dissolution, but it should also impact the kinetics and mechanisms.^54^

It is understood that during the oxidation of Pt surfaces, as certain coverages of adsorbed oxygen (O_ad_) are reached, the so called place‐exchange mechanism begins.^28^, ^29^, ^42^, ^55^, ^56^ During place‐exchange, Pt surface atoms exchange place with O_ad_, and these extracted Pt atoms may be susceptible to dissolution. The exact mechanism(s) of Pt dissolution is yet to be elucidated; however, it is accepted that it is closely related to the mechanism(s) of irreversible place‐exchange during oxidation and reduction,^6^ which could be inherently different on the basal Pt facets.

Determining the mechanism of surface oxidation and reduction is very difficult, as most in situ spectroscopic techniques cannot distinguish between oxygen and hydrogen species adsorbed/absorbed on or in the near surface from the bulk material and the near surface electrolyte. However, recently in situ shell‐isolated nanoparticle‐enhanced Raman spectroscopy (SHINERS)^41^ has shed light on the difference in oxidation mechanisms for Pt(111) and Pt(100).

Through in situ SHINERS, large differences in the intermediate stages of Pt oxidation and oxide growth in 0.1 M HClO_4_ were observed between Pt(111) and Pt(100) facets.^41^ For both Pt(111) and Pt(100) the perchlorate anion initially interacts with an adsorbed hydroxyl phase. For Pt(111) at potentials above 1.1 V_RHE_ a large peak is seen in the CV, and a surface hydroxyl phase is converted to a 2D (su)peroxo‐like surface oxide. Raising the potential further above 1.3 V_RHE_ this 2D (su)peroxo‐like oxide is converted to an amorphous 3D α‐PtO_2_. However, for Pt(100) both the formation of the 2D (su)peroxo‐like surface oxide and amorphous 3D α‐PtO_2_ take place together at potentials above 1.0 V_RHE_. Furthermore, it is speculated that this behaviour of Pt(111) is uniquely different than that of the other facets, as the spectral features of Pt(poly) is similar to that of Pt(100). These results indicate differences in the oxidation mechanism between these two surfaces, which could be related to the differences in dissolution onset and quantities observed here. Unfortunately such data are unavailable for Pt(110) or annealed Pt(poly).

If the coordination number of the well‐ordered single crystal surface atoms is considered, the trend in dissolution is rational, as the coordination numbers of the Pt basal planes are in a sense similar to the under‐coordinated defects present on Pt(poly) or Pt nanoparticles. It is excepted that under‐coordinated defect sites are highly susceptible to dissolution on Pt(poly)^57^ and also on Pt nanoparticles.^58^ A practical example of this was recently shown, in which modifying the surface of Pt nanoparticles with ionic liquids that preferentially adsorb at defect sites significantly decreased Pt dissolution under a variety of electrochemical conditions.^58^ Therefore it can be predicted that the dissolution of the Pt basal planes should follow Pt(111)<Pt(100)<Pt(110) under most conditions, which is the trend observed here.

The surface of Pt(poly) is made up of the Pt basal planes, higher index facets and includes many grain boundaries. Grain boundaries can act as preferential sites for oxidation and dissolution.^59^ Therefore, it is difficult to predict the quantity of dissolution and onset potential relative to Pt(111), Pt(100) and Pt(110). However, it is reasonable to assume that Pt(poly) would have a behaviour in between the basal planes. During CVs and potential holds≥1.2 V_RHE_, Pt(poly) and Pt(100) dissolve similarly, while during holds at 1.0 V_RHE_ Pt(poly) dissolves much more than Pt(100) (only 5 μML at 60 s hold, Figures S3 and S4). It is possible that the low coordinated grain boundary defect sites on Pt(poly) are more susceptible to dissolution at low potentials, and therefore dissolution is seen as Pt(poly)>Pt(100) during holds at only 1.0 V_RHE_. During holds at≥1.2 V_RHE_ the increased potential triggers increased Pt(100) dissolution and the quantities become similar. Dissolution experiments on a polyoriented Pt single crystal bead would eliminate the parameter of grain boundaries and could aid in the understanding of the above observations; however, further development of the SFC design will be required for these future experiments.

The anodic to cathodic dissolution ratios in Figure 5 can be used as an indication of the severity of dissolution during Pt oxidation and reduction. At 1.2 V_RHE_ holds, a general trend in the ratio is seen with Pt(100)>Pt(111)>Pt(poly)>Pt(110). A higher ratio may reflect the dissolution energetic barriers associated with oxidative place‐exchange at anodic potentials and diffusion back into the crystal during reduction. It is possible that the relatively low coordinated surface of Pt(110) has a larger barrier for place‐exchanged Pt atoms to diffuse back into the surface during reduction, and/or that these place‐exchanged atoms are highly unstable, in contrast to Pt(111) and Pt(100). Although these data suggest large differences in the place‐exchange mechanism and dissolution, it is difficult to speculate on such processes while supporting literature on all surfaces is currently lacking.

Potentiostatic holds at 1.4 V_RHE_ yield much different results. The ratios shrink compared to holds at 1.2 V_RHE_, which is to be expected. As the surface becomes passivated by PtO_x_, dissolution slows and will eventually diminish to rates below the detection limit of ICP‐MS. Cathodic dissolution depends on the amount of PtO_x_ formed, which is much greater at increased potentials. The above trend no longer persists, and the large differences could be related to the increased roughening at this higher potential, which is known to develop different morphologies for each surface.^21^, ^54^, ^60^, ^61^, ^62^ The resulting cathodic dissolution would then likely proceed much differently depending on the defects of the varying morphologies with different steps, kinks and terrace facets.

Comparing the new results to those of Lopes et al.,^44^ the most directly comparable experiment is that of the OER, which employs an identical scan rate and electrolyte. During the anodic scan they found the dissolution trend to be Pt(100)≫Pt(111)≈Pt(110) at potentials >1.2 V_RHE_. The reason for such differences is unknown, and the trend is rather unexpected due to the surface energies and coordination of the basal planes. However, differences during crystal preparation could possibly play a role.

Here the flame annealed crystals were cooled for 2 mins in a stream composed of Ar/H_2_ with ca. 40 % H_2_, while the previous work cooled for 10 mins in Ar/H_2_ with 3 % H_2_. The ratio of Ar/H_2_ has been shown to have a profound effect on obtaining a highly ordered Pt(100) surface, where it was suggested that the more H_2_ the better (up to 50 %).^36^ The longer cooling times, and unavoidable exposure to atmospheric O_2_ during transfer to the electrochemical set up can also degrade the annealed surface due to oxidation. When the SFC‐ICP‐MS or SPRDE‐ICP‐MS make electrolyte contact to the crystal, a so‐called contact dissolution peak is observed, the intensity of which depends on surface oxidation from air exposure.^63^ Here the contact peaks (Figure S6) were found to be ca. an order of magnitude lower than those reported for Pt(111) by Lopes et al., suggesting different extents of surface oxidation which can degrade the annealed surface.

The study here clearly shows large dissolution differences between the Pt basal planes, which can be rationalized by distinct surface energies and surface atom coordination. The results hint at differences in oxidation/reduction place‐exchange mechanisms and put further emphasis on the need for increased understanding of these fundamental processes. Advanced experimental in situ electrochemical characterization techniques such as surface x‐ray diffraction (SXRD) and SHINERS will be invaluable in the elucidation of place‐exchange oxidation/reduction mechanisms of platinum crystal facets.^40^, ^41^, ^42^ When combined with modern computational methods illustrating possible low energy barrier pathways,^55^, ^56^ significant scientific advancements are within grasp. Such advances would shed light on the dissolution mechanisms and aid in the knowledge driven development of highly active and stable Pt based catalysts.

The differences in dissolution observed here may also correlate to the stability towards dissolution of preferentially shaped Pt or Pt alloy nanoparticles, which show great promise in terms of activity.^19^, ^20^, ^21^, ^22^, ^23^, ^24^, ^25^, ^26^ It could be hypothesized that octahedral shaped nanoparticles exposing Pt(111) facets dissolve less than cubic shapes exposing Pt(100). However, considering that properties of nanoparticles can drastically differ from bulk materials, dedicated investigations are required.

## 4 Conclusions

It has been shown that the general trend in dissolution for the studied surfaces follows Pt(110)>Pt(poly)≈Pt(100)>Pt(111) and from a thermodynamic perspective is rationalized by the differences in surface energies in which reported values show Pt(110)>Pt(100)>Pt(111). This reflects the coordination of the surface atoms of the basal planes: 7, 8 and 9 for Pt(110), Pt(100) and Pt(111), respectively. Low‐coordinated surface sites are highly susceptible to platinum‐oxide formation, reduction and dissolution and conceivably contribute to differences in the corresponding place‐exchange mechanism(s) which dictate the kinetics of these processes. Potentiostatic holds at the relatively low potential of 1.0 V_RHE_ also caused dissolution for Pt(poly)>Pt(100), which can be due to grain boundaries which act is preferential sites for dissolution. Recent advanced in situ techniques which combine electrochemistry with spectroscopic characterization are beginning to shed new light on the oxidative and reductive processes on the platinum basal facets. However, further efforts are needed to extend the knowledge from Pt(111) to the other crystal planes in order to elucidate the mechanistic differences responsible for trends in dissolution.

## Experimental Section

Electrochemical measurements were carried out in a flow of argon purged 0.1 M HClO_4_ solution using ultrapure water (18.2 MΩ ⋅ cm, PureLab Plus System, Elga) with 70 % Suprapur® perchloric acid (Merck) on the previously described SFC‐ICP‐MS setup.^9^, ^46^ The flow rate of the SFC‐ICP‐MS was ≈170 μL ⋅ min^−1^ and 10 μg ⋅ L^−1 187^Re was used as an ICP‐MS (NexION 300X, Perkin Elmer) internal standard. The working electrode cell contract area was 0.035 cm^2^.

A saturated Ag/AgCl (3 M, Metrohm) and carbon rod (Pentel Hi‐polymer® HB) were used as reference electrode (RE) and counter electrode (CE), respectively, while cylindrical Pt(111), Pt(100), Pt(110) and Pt(poly) crystals with dimensions of 3 mm height and 5 mm diameter were used as working electrodes (MaTeck). The crystal surfaces had an orientation accuracy of <0.1 degrees and roughness <0.1 μm. To make electrical contact for both bulk cell and SFC‐ICP‐MS measurements, Pt wires (Goodfellow Cambridge Ltd., 99.99 %) were soldered onto the backside of the crystals. Prior to any SFC‐ICP‐MS measurement the working electrode crystals (including Pt(poly)) were flame annealed with a butane torch until glowing red hot for at least 30 s, cooled in a flow of Ar/H_2_ (ca. 40 % H_2_) for 2 min and the surface was then protected by a drop of Ar/H_2_ saturated ultrapure water for transfer to the SFC‐ICP‐MS. Such preparation is known to produce highly ordered surfaces in liquid electrolyte studies.^64^, ^65^, ^66^ All potentials reported are against the reversible hydrogen electrode (RHE), which was measured at the beginning of each day. All gasses were supplied by Air Liquide (5.0 purity).

The quality of the single crystal surfaces was confirmed in a custom made glass bulk cell. A Pt wire purged in a separate compartment with H_2_ served as a RHE for the RE. For CE, a Pt wire was placed in the main compartment with the WE. The 0.1 M HClO_4_ electrolyte was initially purged with Ar for at least 30 min, and afterwards the atmosphere was continually purged with Ar.

Dissolved quantities expressed in mML (milli‐monolayers) were calculated using a lattice constant of 3.92 Å for the basal planes and a charge of 210 μC ⋅ cm^−2^ for Pt(poly).^67^ The resulting surface atomic densities of 1.31 ⋅ 10^15^, 1.30 ⋅ 10^15^, 9.19 ⋅ 10^14^ and 1.49 ⋅ 10^15^ atoms ⋅ cm^−2^ for Pt(poly), Pt(100), Pt(110) and Pt(111), respectively, are used to convert between mass per area and MLs. All dissolution measurements were repeated 2–3 times.

Figure S7 illustrates the process of making contact with the SFC‐ICP‐MS setup to the single crystal working electrode protected by a droplet of Ar/H_2_ saturated ultrapure water. The droplet protected crystal was placed beneath the SFC, in a custom made PTFE holder and potential held at 0.07 V_RHE_. The stage was then slowly raised and as contact was made between the droplet and SFC meniscus, with careful control of the working parameters, the droplet was sucked into the cell as contact was established and maintained at 400 mN pressure with a force sensor (KD45, ME‐Meßsysteme). During the process the WE (working electrode) area is always protected by water/electrolyte, and only liquid from outside of the contact area is removed.

## Conflict of interest

The authors declare no conflict of interest.

## Supporting information

As a service to our authors and readers, this journal provides supporting information supplied by the authors. Such materials are peer reviewed and may be re‐organized for online delivery, but are not copy‐edited or typeset. Technical support issues arising from supporting information (other than missing files) should be addressed to the authors.

Supplementary
