# Supplementary material for: Dissolution of Platinum Single Crystals in Acidic Medium
Source: Chemphyschem. 2019 Nov 8;20(22):2997–3003. doi: 10.1002/cphc.201900866 (PMC6899853; doi:10.1002/cphc.201900866)
Supplement: Supplementary file 1 — Supplementary [file CPHC-20-2997-s001.pdf]

CHEMPHYSCHEM

Supporting Information

© Copyright Wiley-VCH Verlag GmbH & Co. KGaA, 69451 Weinheim, 2019

Dissolution of Platinum Single Crystals in Acidic Medium

Daniel J. S. Sandbeck,* Olaf Brummel, Karl J. J. Mayrhofer, Jörg Libuda, Ioannis Katsounaros, and Serhiy Cherevko*©2019 The Authors. Published by Wiley-VCH Verlag GmbH & Co. KGaA. This is an open access article under the terms of the Creative Commons Attribution License, which permits use, distribution and reproduction in any medium, provided the original work is properly cited.An invited contribution to a Special Issue on Electrocatalysis

Table of Contents:

Figure S1 2
Figure S2 2
Figure S3 3
Figure S4 4
Figure S5 5
Figure S6 5
Figure S7 6

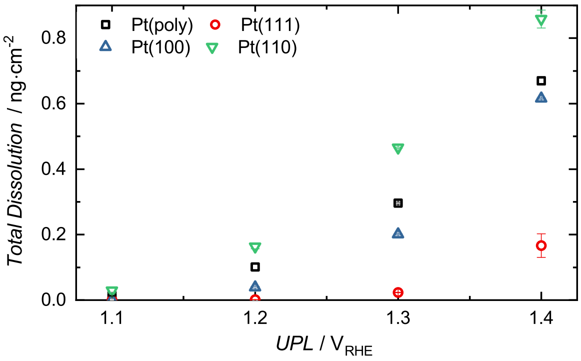

Figure S1: Total quantity of Pt dissolved during CVs to varying UPLs from 0.07 V_{RHE} at 50 $mV\cdot s^{-1}$. Units are shown as mass per geometric area.

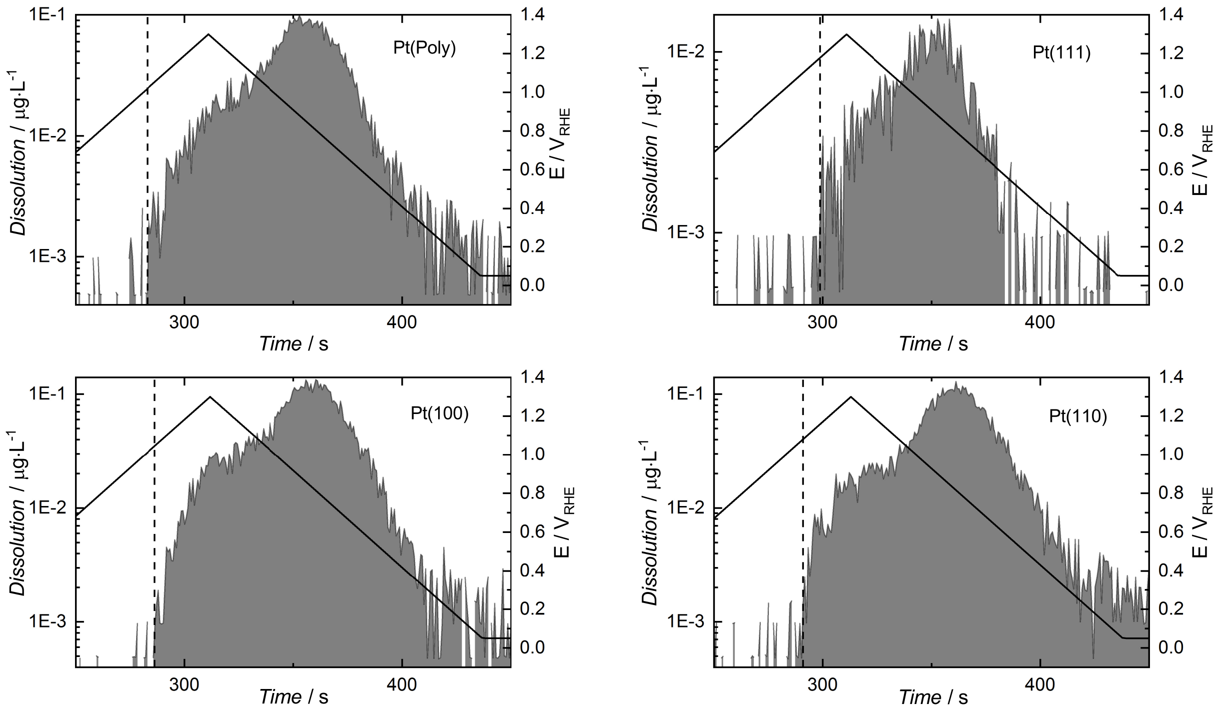

Figure S2: Example of how the dissolution onset potential was estimated. From a 10 mV·s^{-1} CV, a line is drawn from where the dissolution signal increases over the baseline signal. A logarithmic Y-axis (left) is used for the dissolution signal to clearly see the increase in signal. The line from dissolution onset is matched to the corresponding potential on the second Y-axis (right). The time delay between the working electrode and the ICP-MS (from the connecting tubing) was accounted for and subtracted from the dissolution time axis. This was repeated 3 times for each surface.

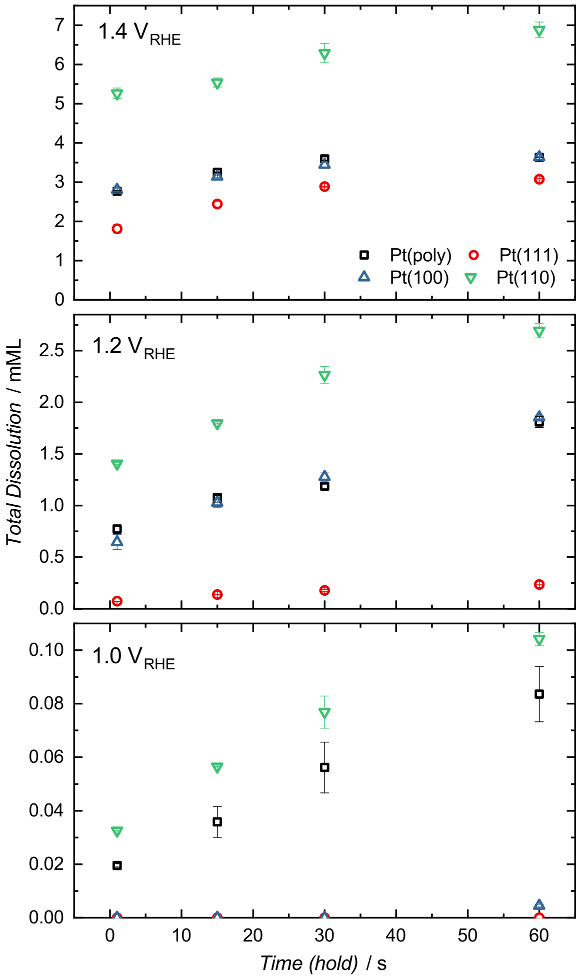

Figure S3: Total quantified dissolution in mMLs (milli-monolayers) during potentiostatic holds at 1.0, 1.2 and 1.4 V_{RHE} for 1, 15, 30 and 60 s.

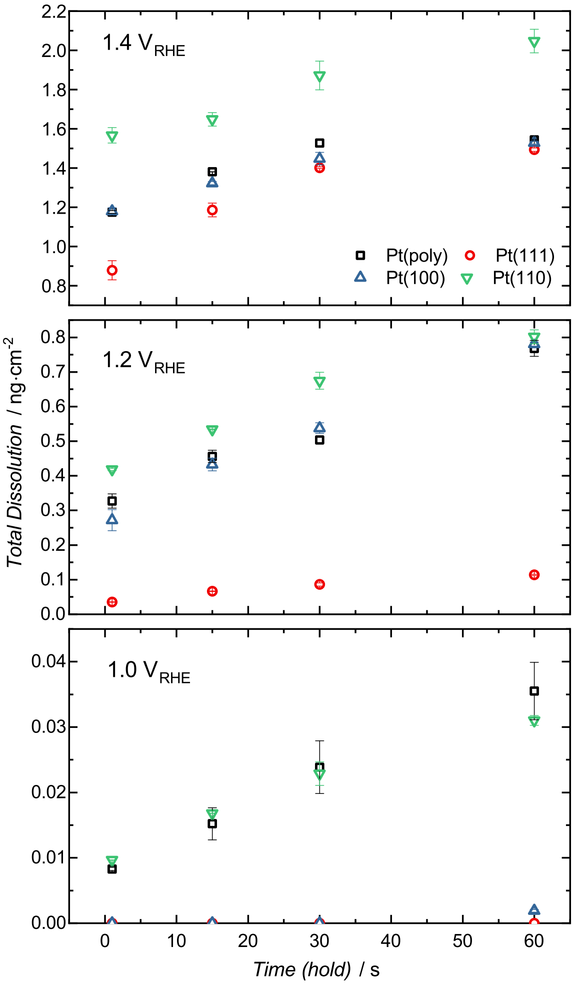

Figure S4: Total quantified dissolution in ng·cm^{-2} (mass per area) during potentiostatic holds at 1.0, 1.2 and 1.4 V_{RHE} for 1, 15, 30 and 60 s.

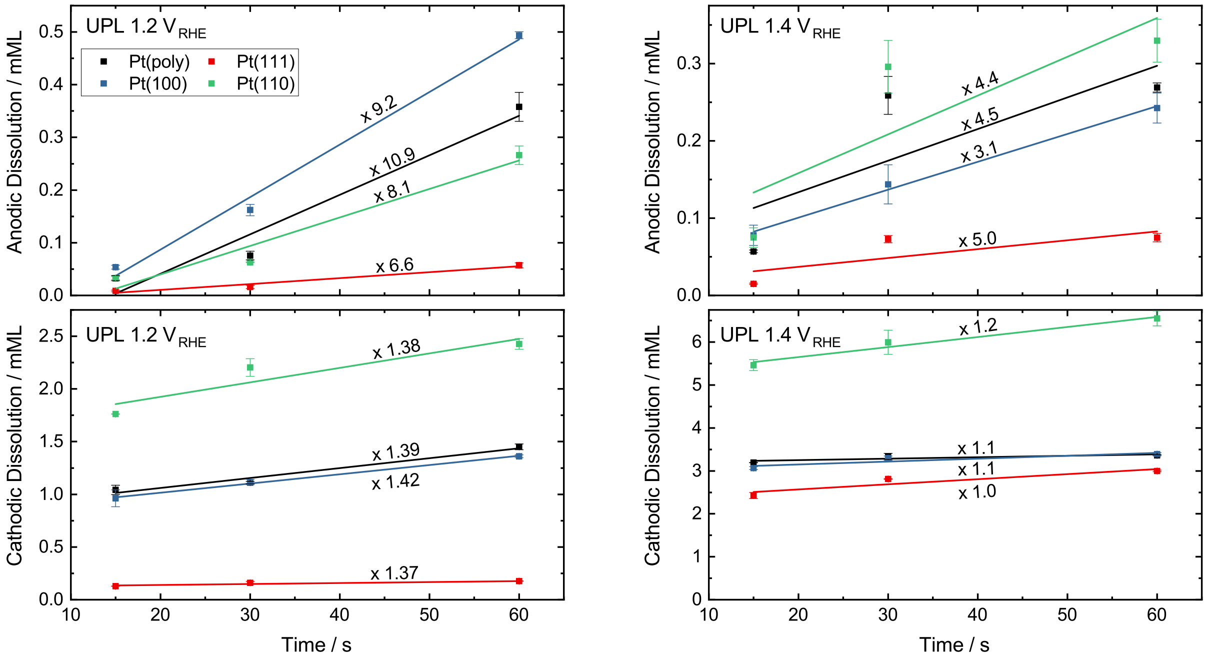

Figure S5: Total dissolved quantities during anodic holds at 1.2 V$_{RHE}$ (left) and 1.4 V$_{RHE}$ (right) and subsequent cathodic potential sweep (50 mV·s^{-1}). Linear fits are shown to help aid the eye, while the multiple increase from the 15 to 60 s hold is also marked.

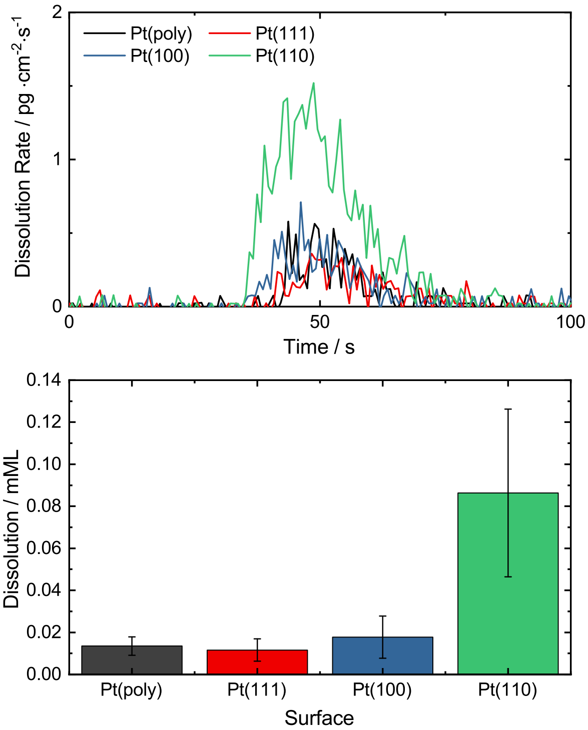

Figure S6: Contact peaks from the SFC-ICP-MS making contact with the droplet-protected annealed WEs at 0.07 V$_{RHE}$ during the CV dissolution experiments.

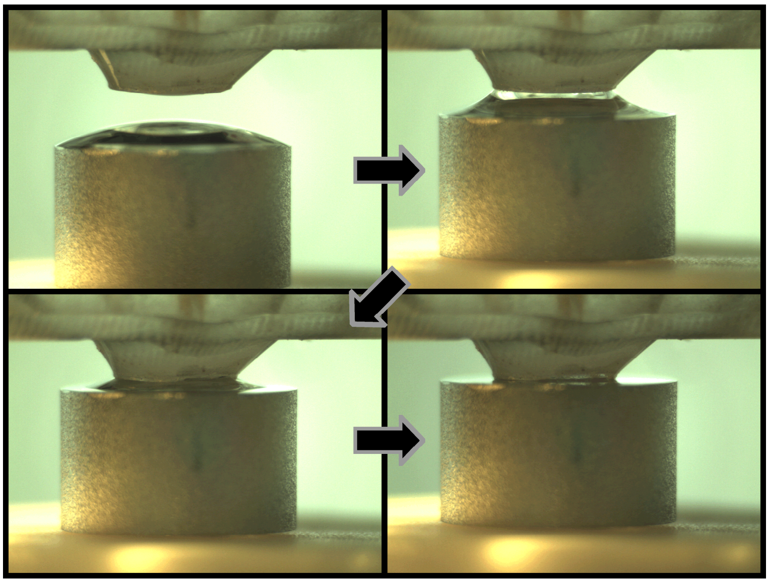

Figure S7: Illustration of the SFC-ICP-MS making contact with a Pt single crystal working electrode
